# Recalling the Future: Immunological Memory Toward Unpredictable Influenza Viruses

**DOI:** 10.3389/fimmu.2019.01400

**Published:** 2019-07-02

**Authors:** Maria Auladell, Xiaoxiao Jia, Luca Hensen, Brendon Chua, Annette Fox, Thi H. O. Nguyen, Peter C. Doherty, Katherine Kedzierska

**Affiliations:** ^1^Department of Microbiology and Immunology, Peter Doherty Institute for Infection and Immunity, University of Melbourne, Melbourne, VIC, Australia; ^2^Research Center for Zoonosis Control, Hokkaido University, Sapporo, Japan; ^3^WHO Collaborating Centre for Reference and Research on Influenza, Peter Doherty Institute for Infection and Immunity, Melbourne, VIC, Australia; ^4^Department of Immunology, St. Jude Children's Research Hospital, Memphis, TN, United States

**Keywords:** T cells, B cells, influenza, immunological memory, vaccine

## Abstract

Persistent and durable immunological memory forms the basis of any successful vaccination protocol. Generation of pre-existing memory B cell and T cell pools is thus the key for maintaining protective immunity to seasonal, pandemic and avian influenza viruses. Long-lived antibody secreting cells (ASCs) are responsible for maintaining antibody levels in peripheral blood. Generated with CD4^+^ T help after naïve B cell precursors encounter their cognate antigen, the linked processes of differentiation (including Ig class switching) and proliferation also give rise to memory B cells, which then can change rapidly to ASC status after subsequent influenza encounters. Given that influenza viruses evolve rapidly as a consequence of antibody-driven mutational change (antigenic drift), the current influenza vaccines need to be reformulated frequently and annual vaccination is recommended. Without that process of regular renewal, they provide little protection against “drifted” (particularly H3N2) variants and are mainly ineffective when a novel pandemic (2009 A/H1N1 “swine” flu) strain suddenly emerges. Such limitation of antibody-mediated protection might be circumvented, at least in part, by adding a novel vaccine component that promotes cross-reactive CD8^+^ T cells specific for conserved viral peptides, presented by widely distributed HLA types. Such “memory” cytotoxic T lymphocytes (CTLs) can rapidly be recalled to CTL effector status. Here, we review how B cells and follicular T cells are elicited following influenza vaccination and how they survive into a long-term memory. We describe how CD8^+^ CTL memory is established following influenza virus infection, and how a robust CTL recall response can lead to more rapid virus elimination by destroying virus-infected cells, and recovery. Exploiting long-term, cross-reactive CTL against the continuously evolving and unpredictable influenza viruses provides a possible mechanism for preventing a disastrous pandemic comparable to the 1918-1919 H1N1 “Spanish flu,” which killed more than 50 million people worldwide.

## Introduction

Successful vaccination relies on the induction of long-term immunological memory. Exposure to an infectious virus elicits acute effector responses that mediate acute pathogen control, along with the generation and maintenance of T cell and B cell memory capable of protecting against re-exposure. At sufficient levels, neutralizing antibody (Ab) can prevent re-infection while, especially if such protection is partial, the rapid recall of memory CD8^+^ cytotoxic T lymphocytes (CTLs) facilitates enhanced pathogen control. Seasonal influenza results from the emergence of an occasional, highly infectious variant selected as a consequence of Ab-driven mutational change in the viral envelope hemagglutinin (HA) and/or neuraminidase (NA) proteins. Pandemic influenza A viruses, on the other hand, arise from gene reassortment of two different influenza A virus (IAV) subtypes infecting the same cells. As a consequence, the influenza research and control community face the continuing challenge of producing new vaccines to control emerging threats.

Most of the existing products utilize inactivated virus, or isolated viral HA and NA proteins, that stimulate influenza strain-specific antibody immunity and B cell memory, but do not prime the much more cross-reactive CD8^+^ CTL compartment. The challenge is thus to add a T cell-targeted vaccine component that promotes CTL memory for the rapid recall of anti-viral CTL effectors to the respiratory tract for early virus control and/or induce cross-protective B cells. In this review, we focus on the nature of optimal memory B cell and T cell generation and ask how we might use this knowledge to overcome the limitations of seasonal influenza vaccines by developing feasible strategies for both inducing and maintaining long-term, cross-reactive immunological memory.

### The Burden of Seasonal Influenza

Seasonal influenza virus is a global health problem. In the United States, influenza virus infections causes 9.2–35.6 million cases of illness, 140,000–710,000 hospitalizations and 12,000–56,000 deaths per year (1). Globally, it is estimated that every year 290,000–650,000 respiratory deaths are due to seasonal influenza (2). The World Health Organisation (WHO) recommends annual influenza vaccination for people at high risk of developing severe disease, and for those in contact with high-risk individuals. Vulnerable groups include the elderly (>65 years), young children (6–59 months), Indigenous populations, patients with chronic medical conditions, pregnant women, and health-care workers (3). National health authorities in the countries with an advanced public health system recommend annual vaccination for everyone 6 months of age and above, both to protect individuals and to limit the spread of the virus through the community (4, 5).

### Influenza Virus Evolution Poses a Challenge for Long-Term Humoral Immunity and Vaccine Effectiveness

Influenza viruses attach to host cells via HA binding to cell surface sialic acids (6, 7). Protective antibodies (Abs) block virus attachment by binding to the antigenic sites (8–11) proximate to the sialic acid receptor binding pocket on the HA head. Such Abs are the best correlate for influenza immunization and are measured using the hemagglutination inhibition (HAI) assay, which detects Abs blocking the capacity of the virus to agglutinate red blood cells by binding to sialic acids on their surface (12). The influenza virus RNA polymerase lacks proof-reading function, with the consequence that there is a constant emergence of mutants (affecting viral fitness and/or immune recognition) carrying substitutions that arise randomly across the genome. Antibody-mediated immune pressure drives the selection of viruses expressing variant HAs and NAs (13, 14) that, if their “fitness” is not unduly compromised, have the potential in nature to cause the process that has long been called antigenic drift (15, 16). Clearly, for a drifted strain to emerge as a clinical problem, its HA must be sufficiently changed to escape neutralization by pre-existing antibodies induced broadly in human populations by past infections and/or vaccinations. The reality that individuals who were once protected are now at risk from the new variant strain is the basis for frequently reformulating seasonal influenza vaccines (17). In contrast, through the process of antigenic shift, influenza viruses incorporate a completely new HA or NA (18), which adds a new virus into the epidemiological mix. When it comes to antibody-mediated selection, the A/H3N2 strains have consistently shown the greatest antigenic drift for the three types of influenza viruses that co-circulate globally and cause seasonal epidemics (A/H1N1, A/H3N2, and influenza B viruses) (16, 19). In general, more extensive epidemics (with increased morbidity and mortality) occur when a novel, seasonal A/H3N2 drifted strain emerges (16, 20, 21).

Multi-component influenza vaccines are designed to elicit serum antibodies against the HAs of one A/H1N1 strain, one A/H3N2 strain and one (or two) influenza B viruses (Yamagata or Victoria) (22). Increased antibody titres induced by vaccination decrease the risk for infection caused by any strains antigenically similar to those included in the vaccine (23, 24), although they confer limited or no protection against other types or subtypes (including drifted variants) of influenza (25). The global WHO network closely monitors the circulation of influenza viruses in humans and other species, including birds, across the northern and southern hemispheres, whereby information derived from the antigenic and genetic characterization of these strains, along with epidemiological data, is used to select the strains to be incorporated into an upcoming seasonal vaccine (26). This strategy can fail, at least in part, as vaccine preparation takes at least 6 months and the product may no longer match all 3 (or 4) circulating viruses by the time it is released (27). Moreover, pre-existing immunity in humans can be highly variable due to age and prior exposures via infection and/or vaccination (28–34). The level of pre-existing human immunity is considered but often difficult to interpret due to high heterogeneity. First-infection ferret antisera is used to identify and characterize new influenza strains, yet repeated exposures to A/H3N2 variants affect Ab quantity and quality, which makes vaccine-strain selection even more challenging (35). Both immunological responses to influenza viruses and influenza vaccine effectiveness are undoubtedly affected by the combination of antigenic drift and prior immunity. Influenza virus evolution has been widely studied, yet it is still largely unknown how cross-reactive B cell memory impacts on Ab responses to new strains.

### B Cell Memory and Imprinting Against Prior Strains

The idea that immunological memory could impact negatively on Ab responses to novel influenza strains first emerged in the early 1950s, when Francis and Davenport observed that the exposure to a new influenza strain induced higher titres of Abs against variants encountered in childhood than against the prevailing strain (36–39). They proposed the colorfully named concept of “original antigenic sin” (OAS), which states that Abs generated against the first antigen (Ag) encountered in childhood would be repeatedly and preferentially induced at every exposure, even if the epitope remained as a minor secondary antigen. This was considered to be sinful, i.e., detrimental for protection against following influenza infections, since the Abs induced poorly neutralized the most recent strain that had actually triggered them (40, 41). Molecular level analyses of B cell receptor usage have since confirmed that memory B cells elicited by a priming Ag can participate in the immune response toward a structurally related, boosting Ag (42, 43). While it is clear that somatic mutation of the immunoglobulin (Ig) variable (V) region takes place, the extent to which this leads to increased affinity for the priming vs. boosting variant remains controversial (42). These molecular analyses are consistent with more recent observations that Ab boosting is broad, and greatest against more similar viruses, differing somewhat from the OAS concept that centers on the initial antigen encountered (44, 45). Efforts to understand why prior vaccination enhances vaccine effectiveness in some influenza seasons, yet attenuates it in others, has led to further refinements to the OAS hypothesis, namely that imprinted B cell memory responses are not inevitably “sinful” i.e., ineffectual (31). Hensley et al. propose that Ab become focused on selected epitopes which are relatively conserved between successive strains due to a form of competitive dominance by memory B cells and that while this may result in high Ab titres and clinical benefit it may, alternatively, compromise protection if the epitope is altered in future strains. This hypothesis is based on molecular and serological analyses that document focused HI Ab responses in selected individuals (29, 30, 46–49).

At the cellular level, it is clear that memory B cells respond more rapidly than their naïve precursors. Hence, antibody responses may become focused on epitopes that were present in earlier strains because memory B cells specific for those epitopes become rapidly activated at the expense of naïve B cells, which need a higher threshold to respond (50, 51). Memory B cells that bear affinity matured antigen receptors may also be better able to compete with existing Abs for inducing antigen than naïve B cells (52). Several strategies have the potential to promote naïve B cell activation and broaden the Ig response. These include giving repeated vaccine doses (39), increasing the amount and concentration of antigen (53), and adding adjuvants (54). Another suggested mechanism that may promote the enhanced engagement of memory (vs. naïve) B cells is that T regulatory cells (T_regs_) induced by the initial encounter reduce the amount of antigen presented on dendritic cells, thus diminishing the antigen availability for naïve B cells, promoting a memory B cell boost at the expense of naïve B precursors (55).

### Current Strategies to Improve Seasonal Influenza Vaccine Effectiveness

Strategies to increase seasonal influenza vaccine effectiveness (VE), like high-dose or adjuvanted vaccines, are still under evaluation. Pooled analysis of multiple studies showed that high-dose vaccines significantly reduce the risk of laboratory-confirmed influenza cases in the elderly when the vaccine and the circulating strains are well-matched, but not when they are mismatched. The HAI geometric mean titres after vaccinating with the high-dose vaccine were significantly higher compared to the standard-dose vaccine for the H3 component. However, the proportion of participants with seroprotective HAI Ab levels (HAI titer ≧ 1:40 or 1:32) was the same using both vaccines (56). Similarly, high-dose vaccines showed significant increases in VE with a reduction in mortality among the elderly by 36.4% in the 2012–2013 season, when H3N2 viruses were predominantly circulating (57). Nonetheless, seasonal VE on that season was only of 11% for that particular age group (58), indicating that a high-dose vaccine, despite increasing VE, did not induce an epidemiologically significant improve in overall H3N2 VE. Alternatively, the use of a standard-dose influenza vaccine with the MF59 adjuvant (Novartis) can reduce laboratory-confirmed influenza cases as well as hospitalizations due to influenza in the elderly (59) and seasonal trivalent vaccines formulated with this adjuvant are now available for those >65 years old (FluAd, Sequiris).

In addition to MF59, other adjuvants licensed for use with inactivated or sub-unit-based influenza vaccines include Alum-containing formulations (AlPO_4_ or Al[OH]_3_) and oil-in-water emulsions, AS03 (GSK) and AF03 (Sanofi Pasteur). The benefits of using these adjuvants to increase seroprotective antibody titres are widely reported in a number of clinical studies, including in individuals who are most susceptible to influenza-related illness. Compared to non-adjuvanted vaccine responses, formulation of mono- and multi-valent influenza vaccines with MF59 induces substantially higher HAI titres and seroconversion rates in children (60–63) with similar improvements observed in the young and elderly using AS03 (64). These formulations are generally well-tolerated and safe, however, incidences of narcolepsy associated with the use of an AS03-adjuvanted A/H1N1pdm2009 vaccine (Pandemrix) limits the use of this adjuvant in the young. Nevertheless, both MF59 and AS03 have been shown to accelerate the induction of vaccine-mediated responses as demonstrated by the use of adjuvanted vaccines in healthy adults (65, 66), children (67) and in the elderly (68), wherein a single vaccination dose is sufficient to induce seroprotective levels of antibody within as little as 3 weeks. In this regard, these adjuvants, along with AF03 or Alum, provide dose-sparing capabilities for mass vaccination of the wider population; similar levels of protection attained with unadjuvanted vaccines can be achieved with using substantially smaller amounts of HA antigen or less vaccination doses when formulated with adjuvant (69–72). Several studies have also demonstrated the ability of MF59 to induce cross-reactive antibodies against non-vaccine matched strains in prime-boost regimens. Priming of subjects with a clade 0 H5N3 vaccine formulated with MF59 followed by a boost with a clade 1 H5N1 vaccine containing the same adjuvant results in high titres of cross-neutralizing antibody against H5N1 clade 0, 1 and 2 viruses (73–75). These results thus highlight the role that adjuvants can play in generating and broadening the cross-specificity of naïve and pre-existing B cell memory, the possible underlying mechanisms of which are discussed further in subsequent sections below.

Influenza vaccines designed to target Abs toward the conserved epitopes in the HA stem are also under intense study. While heterosubtypic protection with group 1 HA stem vaccines (i.e., H1 and H5 viruses) lacking the highly variable HA head has been demonstrated in animal models (76), studies on group 2 HA stem vaccines (i.e., H3 and H7 viruses) are more limited. Although promising results are observed when immunizing mice with conserved HA stem epitopes from the H3 subtype, by way of cross-clade neutralizing activity (77, 78), immunogenicity and protection are not maintained when using larger animal models like ferrets (78). Therefore, further studies are needed to develop a human B cell-based universal influenza vaccine, with consideration into the potential for influenza viruses to escape from HA-stem targeted Abs (79).

### Dissecting the B Cell Response

Activation of naïve B cells can elicit short-lived ASCs (also called plasmablasts), long-lived antibody-secreting plasma cells (LLPCs), and memory B cells. The fate of B cells is considered to be highly orchestrated, depending on the mode of stimulation, the affinity of their B cell receptors (BCR, or surface Ig) for antigen and their location (80–82). In the periphery, within secondary lymphoid organs (SLO), naïve B cells are activated by BCR/Ag binding and, depending on whether T cell help is provided, they will continue the response in a T cell-dependent (TD) or T cell-independent (TI) manner. B cell memory resulting from a TI response expresses and produces IgM capable of engaging at broadly low affinity with antigens via multivalent BCR engagement, plus toll-like receptor (TLR), and/or complement engagement (83). In TD responses, B and T cell interaction occurs when antigen is captured through the BCR of specific naïve B cells and presented via cell-surface MHC-II glycoproteins to CD4^+^ helper T cells specific for peptides from the same antigen (84, 85). All B cells activated in this manner either move into lymph node follicles and generate germinal centers (GCs) or differentiate into extrafollicular plasmablasts (86, 87). Through this array of processes, different classes of memory B cells are generated, which can be distinguished by their passage through the GC, location and Ig isotype (81).

In the GC, B cells undergo intense proliferation and broaden their BCR diversity through somatic hypermutation, a process whereby point mutations, insertions, and deletions are introduced within Ig V gene hotspots to generate a broad array of B cell clones with a broad spectrum of affinities for the immunizing Ag (88). This process results in the generation of memory B cells with high-affinity surface Igs and surface Ig^+/−^ plasma cells that maintain serum immunoglobulin levels against the foreign invader. The GC is also the site where a large proportion of BCR-defined clones undergo class switch recombination (CSR), exchanging the Ig isotypes originally expressed (IgM and IgD) for IgG, IgA, or IgE (88–90). The sequential generation of long-lived memory B cells in the GC starts from unswitched memory B cells, followed by class-switched memory B cells and, finally, by LLPC that travel to the bone marrow and other sites (91). The later each B cell population appears, the higher its affinity for Ag (92). Hence, B cells with lower affinity BCRs have a greater propensity to enter, and persist in, the memory pool. Intriguingly, such memory-directed B cells show enhanced Bach2 transcription factor expression when compared to their counterparts with higher BCR affinity, and Bach2 expression inversely correlates with the strength of the B-T follicular helper (Tfh) cell interaction. This suggests that B cells with lower affinity receive weaker T cell help and express higher levels of Bach2, which is clearly a key factor in memory B cell fate determination (92). In addition, expression levels of Blimp-1, the key regulator of plasma cell differentiation and CSR, are regulated by Bach2. Higher Bach2 levels decrease Blimp-1, promoting B cell differentiation toward an unswitched memory fate. The aryl hydrocarbon receptor (AhR), a ligand-induced nuclear transcription factor, is highly induced in B cells upon BCR engagement. AhR promotes Bach2 expression, which in turn suppresses Blimp-1 and therefore the B-Tfh cell interaction becomes weaker and B cell CSR and differentiation into plasma cells are suppressed (93), indicating that it may be a potential target in promoting the generation of low-affinity IgM^+^ B cell memory upon vaccination. This is of particular relevance for the design of the next generation influenza vaccines since, as discussed below, as there is an increasing body of evidence suggesting that low-affinity IgM^+^ memory B cells capable of identifying a broad range of epitopes should be targeted by influenza vaccination.

#### Heterogenous Memory B Cell Phenotypes Have Different Roles in Secondary Responses

**Figure 1:**
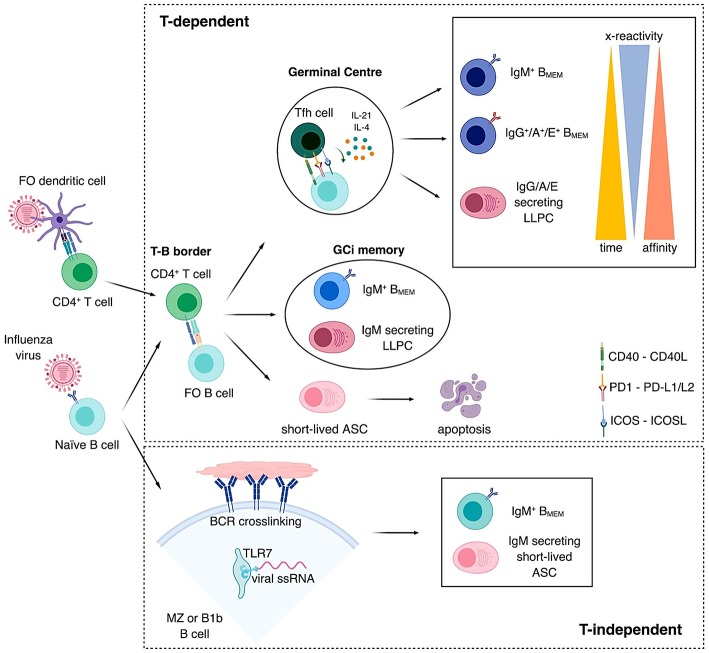
Pathways to B cell memory. Naïve B cells become activated by direct recognition of antigens expressed on the surface of the pathogen. Top panel: Follicular (FO) naïve B cells become activated within the lymph node through a T cell-dependent pathway. CD4^+^ T cells become activated by recognizing viral peptides processed by FO dendritic cells and presented on their surface by MHC-II molecules. After becoming activated, both CD4^+^ T cells and B cells, travel to the T-B border in the lymph node, where they interact. Three outcomes can follow this interaction. (i) A germinal center (GC) is formed, CD4^+^ T cells polarize into T follicular helper (Tfh) cells and FO B cells differentiate into GC B cells. In the GC, B cells undergo rapid proliferation and somatic hypermutation of the Ig V regions in their B cell receptors (BCR), due to their interaction with Tfh cells through CD40-CD40L, PD1-PD-L1/L2, ICOS-ICOSL among others and the secretion of cytokines such as IL-4 and IL-21, affinity maturation takes place and those B cells that increase affinity toward their Ag are selected. Some of these B cells will also class-switch. These interactions result in the generation of IgM^+^ memory B cells (B_MEM_), IgG^+^/A^+^/E^+^ B_MEM_ or IgG/A/E secreting long-lived plasma cells (LLPC) in this order in time. The later these cells are generated, the higher affinity and lesser cross-reactivity they have toward the antigen or antigen variants, respectively. (ii) Not all B cells enter the GC after interacting with their cognate activated CD4^+^ T cells in the T-B border, IgM^+^ B_MEM_ and IgM secreting LLPCs are also generated outside of the GC, in a GC-independent (GCi) manner. (iii) Short-lived antibody secreting cells (ASC) are generated early after activation to generate a rapid response against the pathogen. These short-lived ASC will undergo apoptosis and do not contribute to the generation of B cell memory. Bottom panel: Some protein antigens provide highly repetitive antigenic structures, which induce strong BCR crosslinking. Viral single-stranded RNA (ssRNA) together with other danger signals also activate toll-like receptors such as TLR7. These strong signals are enough to activate B cells in a T cell-independent (TI) manner and generate short-lived IgM secreting ASC and IgM^+^ B_MEM_. B1b and marginal zone (MZ) B cells are activated in a TI manner and provide a faster response against the pathogen.

The various modes of TD and TI B cell activation generate memory B cells with varying isotypes and affinities (summarized in Figure 1), some bearing highly mutated Igs generated via the GC reaction and others maintaining germline, less specific and more cross-reactive Abs (52, 81, 94). While it is generally accepted that memory B cells show an enhanced capacity for terminal differentiation into ASC, regardless of phenotype and affinity, there is less consensus regarding their propensity to (re)-enter GC reactions. Contrary to early thinking, it is now generally accepted that both IgG^+^ and IgM^+^ memory B cells can re-enter GC reactions, albeit they are more predisposed to differentiate into ASC during recall responses (52, 95–97). Similarly, whether or not GCs form during recall responses together with the character of the memory B cell subsets that participate may depend on the type and amount of antigen, inflammatory signals and the availability and quality of cognate Tfh cells (98). There is evidence that unswitched memory B cells bearing germline BCRs have a greater propensity to enter the GC reaction (99). In particular, IgM^+^ cells with the least mutated V genes were more prominent within GCs during the recall response to a variant viral protein antigen rather than to the original inducing antigen when sequentially immunizing mice with variant Dengue envelope proteins with 63% amino acid identity (100). However, when using HAs from more closely related influenza viruses, with ~82% sequence identity, the GC response was dominated by highly mutated memory B cells, which led to a worsened antibody response as compared to the primary encounters, even in the presence of an adjuvant (101). In the elderly, a poor adaptive capacity of B cells toward the drifted influenza epitopes has also been demonstrated. This resulted in the expansion of B cell memory targeting mostly conserved but less potent epitopes (102). In contrast, memory B cell expansion after H3N2 infection reflected imprinting toward strains encountered early in life but also adaptation to the infecting virus (103). These studies suggest that a certain degree of an antigenic difference is needed to induce a protective secondary antibody response by stimulating broadly cross-reactive low-affinity IgM^+^ memory B cells. High-dose and adjuvanted vaccines may improve VE when influenza vaccines strains are antigenically-different. The propensity for IgM^+^ memory B cells to dominate recall GC responses may be further determined by pre-existing antibodies that may outcompete the BCRs from low affinity naïve and IgM^+^ memory B cells, but not high affinity IgG^+^ memory B cells, for antigen (52, 96, 104).

In consideration of the potential for influenza Ab responses to become focused on epitopes present in successive vaccine strains to the detriment of recognizing future variants, it seems appropriate to think in terms of future vaccines that maintain plasticity and heterogeneity within the B cell response. For example, vaccination strategies that recall IgM^+^ memory B cells with less-mutated BCR repertoires, while also inducing naïve populations together with cognate Tfh cell memory to facilitate memory GC formation (98), may tend to skew the overall response toward the generation of more cross-reactive Abs against variant epitopes.

#### T Cell-Independent B Cell Responses Against Influenza

In contrast to TD Ags, which are generally proteins that cannot induce cross-linking of multiple BCRs, TI Ags are generally multivalent polysaccharides or other molecules that contain a repetitive array of antigenic epitopes that have that BCR-polymerization propensity. This paradigm is, however, challenged by the finding that high doses of a monomeric protein Ag can also elicit an exclusive TI B cell response (105, 106). In mouse experiments, both TD and TI B cells give rise to short-lived plasma cells and memory B cells (107–109) and contribute not only to resolving primary influenza virus infection, but also to more effective control of virus replication and symptoms after secondary challenge (110). The recall capacity of TI memory B cells is largely a result of Ag driven clonal expansion, however, like other memory B cells, TI memory B cells are able to respond more readily to Ag than their naïve counterparts.

The capacity of inactivated whole (vs. split) virion vaccines to induce superior influenza virus-specific antibody responses (111–113) may in part be due to the greater induction of TI B cell responses (114). Notably, when TI B cell responses were induced Ab affinity and neutralizing activity was enhanced. The ability of inactivated whole virions to induce TI B cell responses is linked to the presence of single-stranded RNAs that activate B cells via a TLR7-dependent mechanism (114), hence TLR7/8 agonists should be considered as potential adjuvants for seasonal influenza vaccines.

#### Importance of Location for Influenza-Specific Memory B Cells

Unlike LLPCs, memory B cells persist as tissue-resident or circulating among the SLO (115). Memory B cells resulting from a local infection also localize in the affected organs. This occurs following influenza virus infection when influenza-specific memory B cells can be found, not only in lymphoid organs, but also in the lungs. Moreover, memory B cells are also differentially distributed among the lymphoid tissues, indicating that trafficking is influenced by local tissue factors (116, 117). After influenza re-exposure, lung-resident memory B cells differentiate into plasmablasts, providing IgG and IgA *in situ* that quickly neutralizes the virus (117, 118). In general, IgA^+^ memory B cells seem to localize preferentially to the blood and to tissue sites of pathology, while IgG^+^ memory B cells are broadly distributed among tissues that may, or may not, be directly involved in the disease process (116, 117). B cell memory and secreted IgA located in the lungs are essential to provide a quick and effective response against influenza viruses upon exposure, yet current influenza vaccines fail to strongly boost IgA responses (119). Antigen reaching the mucosa of the lung is required to potentially induce stronger IgA responses and for the generation of lung-resident memory B cells, which establish early after infection. The varied location of memory B cells according to their isotype, together with the fact that different environments drive B cell class-switching to a specific isotype, are of particular interest for vaccine design, particularly where (as in influenza) mucosal surfaces are the primary site of infection.

#### T Follicular Helper Cell Memory: Recent Advances in Influenza Vaccination

When the GC contracts, the GC Tfh cells exit and develop into resting memory Tfh cells with a less polarized Tfh phenotype (120–125). Tfh cells with a resting memory phenotype both recirculate in blood and can be found in BM, spleen, and lymph nodes (126–128). Circulating Tfh (cTfh) cells are the most accessible subset in humans. Of increasing research interest, cTfh cells are heterogeneous and can be classified into different subsets based on surface marker expression. Resting cTfh cells express CCR7, which differentiates them from their GC counterparts. When cTfh cells become stimulated, they downregulate CCR7 to traffic to the GC (129). Three different subsets of cTfh cells can be distinguished according to the surface expression of the chemokine receptors CXCR3 and CCR6, which are involved in inflammatory-homing and epithelial and mucosal site-homing, respectively (130, 131). The Tfh1 cells are CXCR3^+^ CCR6^−^, express the T-bet transcription factor and secrete the Th1 cytokine IFNγ. Conversely, the CXCR3^−^CCR6^−^ Tfh2 set expresses the transcription factor GATA3 and produces the Th2 cytokines IL-4, IL-5, and IL-13. Then the Tfh17 cells CXCR3^−^CCR6^+^ cells express the transcription factor RORγT and secrete the Th17 cytokines IL-17A and IL-22 (132).

An overall consensus on the functional implications of the different Tfh subsets regarding B cell help is yet to emerge. While the Tfh1 cells are thought not to be efficient B cell helpers, the opposite is true for the Tfh2 and Tfh17 populations (132, 133). However, human studies on the cTfh response following influenza vaccination demonstrate an increase of circulating, activated cTfh1 cells peaking on day 7 after vaccination that positively correlates with the generation of protective Ab responses and the presence of ASCs in blood (115, 134). In the context of influenza immunization, when culturing human cTfh1 cells isolated at day 7 after priming with either naïve or memory B cells, the cTfh1 cells stimulate memory B cell differentiation into plasmablasts, while naïve B cells remain resting. Yet, naïve B cells cultured with Tfh2 and Tfh17 cells can differentiate into plasmablasts (134). Because Tfh cells are essential to induce a proper B cell response and we speculate that naïve B cells are not being sufficiently stimulated due to epitope masking by pre-existing Abs and memory B cells, it could be possible that mainly Tfh1 cells are stimulated after influenza vaccination at the expense of Tfh2 and Tfh17.

### Anti-viral CD8^+^ T Cell Responses

Seasonal influenza vaccines are designed to elicit an Ab response. However, the natural influenza virus infection additionally elicits cellular immunity (CD8^+^ T cells, CD4^+^ T cells, MAIT cells, NK cells) to eliminate the infection. Because influenza viruses are under constant selective pressure, the long-term protective value of any vaccine that targets a specific HA and/or NA will inevitably be compromised with time, immune CD8^+^ T cells are critical for recovery and provide some protection against severe influenza disease, including that resulting from infection with a previously unencountered avian strain. This likely reflects that influenza-specific CD8^+^ T cells tend to recognize HLA-bound peptides derived from more conserved, internal virus proteins. The question is whether vaccines that promote such CD8^+^ T cell memory can, when combined with the classical products that induce virus-specific Ig response, provide better protection against, in particular, a newly invasive pandemic strain. An overview comparison between B and T cell responses after influenza virus drift and shift and how they complement each other is shown in Table 1.

**Table 1:** The clinical outcome and the B and T cell memory responses after exposure to influenza viruses are summarized below.

	**Influenza antigenic site change**			
	**Antigenic drift** ***Genetic changes in Ag sites alter Ab binding***			**Antigenic shift**
	**None**	**Minimal**	**Major**	**Exchange of surface Glycoproteins**
Clinical outcome	Little to no symptoms		Unpredictable (135)	Dependent of CD8^+^ T cell response •Limited by HLA alleles (136, 197) •Prior exposure to influenza (137–139) T cell memory pool and quality of T cell response (137–139) ⇒ Severe to fatal outcome with prolonged hospitalizations (137)
B cell response	Robust memory B cell response and protective Ab production (135)	Dominated by memory B cells against preserved antigenic sites, yielding a protective but focused Ab response that may not protect against future drift.	Cross-reactive memory B cells produce an early unadapted Ab response to limit virus replication and symptoms, and enter GC reactions to generate updated memory and PCs If enough Ag available, naïve B cells react and generate updated B cell memory	Very limited (if any) protection by memory B cells (31, 140)Response driven by naïve B cells
CD8^+^ T cell response	Cross-reactive Not responsive if B cells neutralize the virus			Cross-reactive but not neutralizing immunityHost-specific differences

Adaptive T cell immunity is mediated primarily by T cells, expressing the CD4 or CD8 co-receptors, respectively. During influenza virus infection, viral proteins are degraded by the proteasome and processed into smaller peptide fragments. These fragments are bound to MHC molecules and carried to the cell surface for presentation. These peptide/MHC complexes (pMHC) are recognized by clonally expressed TCRs on CD4^+^ or CD8^+^ T cells, leading to their activation and recruitment into the virus-specific immune response. The CD8^+^ cytotoxic T lymphocytes act as sentinels, recognizing and killing virus-infected targets, an essential step for virus clearance. Following activation, CD8^+^ T cells also secrete anti-viral cytokines (especially IL-2, IFN-γ, and TNF-α) which further recruit innate and adaptive immune cells into sites of influenza virus-induced pathology and induce anti-viral responses in infected cells (141, 142). When it comes to CTL killing, the secretion of perforin, granzymes and FAS ligand can all be involved in the process of inducing the apoptosis of virus-infected cells (143, 144). Additionally, the expression of TRAIL on CTLs can lead to the elimination of influenza virus infected cells, with a resultant decrease in mortality (145).

#### T Cell Fate: to Die or Become Memory

Formation of memory CD8^+^ T cells is essential for the protection against re-encountered pathogens. Our understanding of key factors determining the fate of CD8^+^ T cells during influenza is still limited but crucial for the development of a CD8^+^ T cell activating vaccine. During differentiation from naïve to effector, to memory status, CD8^+^ T cells transiently express cell surface molecules that are considered to be predictive of cellular fate and function. Surface expression of IL-7R and KLGR1 on effector CD8^+^ T cells can, at least in some situations, differentiate between CD8^+^ T cells designated as memory precursor effector cells and short-lived effector cells (146). Compared to the IL-7R^lo^KLGR1^hi^ set, CD8^+^ T cells expressing high levels of IL-7R and low levels of KLGR1 are 10-fold more likely to survive (147) in mice infected with lymphocytic choriomeningitis virus (LCMV). However, it should be noted that these profiles may not be exclusive, as KLRG1^+^ CD8^+^ T cells are detectable after LCMV infection is cleared (148), and the survival value associated with the IL-7R^hi^KLGR1^lo^ set for LCMV is less obvious for influenza virus infection (149). Additionally, the discovery of other early markers of memory formation during *Listeria monocytogenes* and vesicular stomatitis virus infection, including expression of ID3 transcription factor (150) and IL-2Rα cytokine receptor, showed that CD8^+^ T cell memory generation is certainly multi-factorial (151, 152). Identifying markers of successful memory formation is crucial for evaluation of novel influenza vaccine responses and should be considered in future influenza vaccine studies. More recently, high-throughput sequencing is facilitating the emergence of a broader picture for CD8^+^ T cell differentiation. Single-cell RNAseq of CD8^+^ T cells at the acute phase of LCMV infection indicates that there may be two distinct populations of antigen-induced CD8^+^ T cells that share genes either with “terminal effector” or “memory” cells (153). Compared to naïve CD8^+^ T cells, the “terminal effector-like” set can be shown to have upregulated more than 900 different genes, while the “memory-like” cells only upregulated 27 genes (153). This suggests that the differentiation of “terminal effector” CD8^+^ T cells mandates the upregulation of hundreds of genes involved in both clonal expansion and the mediation of a spectrum of effector functions, while the establishment of CD8^+^ T cell memory requires only the involvement of a few key genes to maintain lymphocyte quiescence**. **Although the exact factors mediating distinct CD8^+^ T cell fates during early division following viral infection are still in the process of elucidation, experiments with TCR-transgenic mouse models indicate that TCR signaling strength (154), as reflected in IL-2R, IFN-γR, and mTOR levels during mitosis and asymmetrical division (155–157) is key to the generation of anti-viral CD8^+^ T cell memory. This is an exciting area of research that should, as it unfolds, give a much better understanding of both the molecular basis of CTL memory formation, and provide key measurement parameters that will allow us to skew early vaccine responses so that they provide optimal memory that gives long-lasting protection when recalled by further pathogen challenge.

#### Importance of Generating Long-Term T Cell Memory

As mentioned above, memory CD8^+^ T cells are important for eliciting long-term, broadly cross-reactive immunity to influenza viruses, and are thought to mediate the protective function mainly via the killing of virus-infected targets (158). Virus-specific CD8^+^ effector T cells also produce proinflammatory cytokines, and the breadth of cytokine production (termed polyfunctionality) often correlates with efficient protection against pathogens, including influenza viruses (159). Polyfunctional memory CD8^+^ T cells (producing IFN-γ, TNF, IL-2, and MIP-1β) (160) are thought to operate via augmented cytolytic activity via dual IFN-γ/TNF expression (161), IL-2-mediated enhancement of CD8^+^ T cell memory function (162) and increased IFN-γ secretion on a per cell basis (163). One example of the protective capacity of these polyfunctional memory CD8^+^ T cells is the induction of long-lasting memory CD8^+^ T cells against variola (smallpox) virus induced by the Vaccinia vaccine Ankara (164). When CD8^+^ T cells were primed with influenza virus nucleoprotein (NP) expressed by either a recombinant Vaccinia virus or in *Listeria monocytogenes*, the more polyfunctional NP-specific CD8^+^ T cells were generated following Vaccinia virus exposure. Mice vaccinated with the Vaccinia virus showed also a greater level of protection against a normally lethal IAV challenge compared to *the Listeria monocytogenes* vaccine group counterparts (165). This indicates, that not only the quantity of memory CD8^+^ T cells is critical for the protection but also their quality. Insights into key factors inducing these polyfunctional CD8^+^ T cells could improve a T cell-based vaccine therefore vastly.

**Figure 2:**
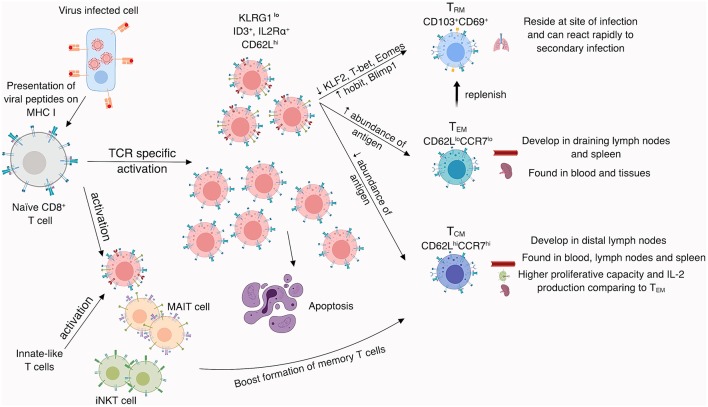
CD8^+^ T cell memory formation. Naïve CD8^+^ T cells become activated by recognition of viral peptides presented in the context of MHC-I molecules on the surface of virally-infected APCs. Activated CD8^+^ T cells divide and differentiate into effector CD8^+^ T cells, which kill virus-infected cells and secrete cytokines to induce an anti-viral milieu. After viral clearance, mainly KLRG1^lo^, ID3^+^, IL2Rα^+^, and CD62L^hi^ CD8^+^ T cells develop into CD8^+^ memory T cells, while the remaining ~90–95% of CD8^+^ T cells undergo apoptosis. Memory formation can be augmented by innate-like T cells (iNKT and MAIT cells). Memory CD8^+^ T cells are divided based on surface marker expression, known to impact their localization. While T_CM_ and T_EM_ can be found in blood and tissues, T_RM_ reside at the site of infection where they can rapidly respond towards a secondary infection. T_CM_ can be also found in lymph nodes and display higher proliferative capacity and IL-2 production compared to their T_EM_ counterparts.

Memory CD8^+^ T cells can be divided conceptually into central and effector T cell memory sets, based on their expression profiles for the CD62L and CCR7 surface proteins (166) that are known to affect cell localization and function (167). The CD62L^hi^CCR7^hi^ “central memory” CD8^+^ T cells (T_CM_) can be found in the spleen, blood and lymph nodes, and display superior functions compared to their CD62L^lo^CCR7^lo^ effector memory CD8^+^ T cell (T_EM_) counterparts, mainly in terms of their proliferative capacity and IL-2 production profiles (168). In addition, a highly specialized population of tissue-resident (T_RM_) memory CD8^+^ T cells expressing CD103^+^CD69^+^ can persist in sites of pathology subsequent to virus clearance (169). Following the secondary challenge, CD103^+^CD69^+^ T_RM_ set is able to expand and secrete cytokines, including IFN-γ and TNF, as well as generate more polyfunctional progeny (69% of cells capable of secreting three cytokines), when compared to CD103^−^CD69^+^ (21%) and CD103^−^CD69^−^ (16%) parent subsets (160, 169). In the context of influenza, persistence of influenza-specific CD8^+^ T_RMs_ correlates strongly with protection when mice are challenged with a serologically distinct IAV that shares common internal proteins (170). The T_RM_ population develops from precursors lacking KLRG1 (171, 172) and further studies on T cell receptor (TCR) repertoires suggest that they arise from the same naïve pool as T_CM_ set (173). T_RM_ generation is largely regulated by a series of transcription factors (174), such as Runx3 which is crucial for T_RM_ establishment across a range of tissues (175), and Bach2 which is recognized to restrain the terminal differentiation of effector T cells and help with formation of long-term memory T cells (176). The differentiated T_RM_ phenotype is associated with changes in key transcription factors, including downregulation of Kruppel-like factor 2 (KLF2), TCF1 (177), T-bet, and Eomes (178, 179) as well as upregulation of Hobit, Blimp1 (177) and AhR (180), Nur77 (181), and Notch (182), required for the maintenance of T_RMs_. While the previously named transcription factors are universal hallmarks of T_RM_ formation, T_RM_ heterogeneity among cells generated at different tissue sites suggest that microenvironmental cues are important for site-specific T_RM_ differentiation. Indeed, generation of the lung T_RM_ set is influenced by transforming growth factor β (TGF-β) along with the presence of IFN-γ-secreting CD4^+^ T cells following influenza virus infection (183, 184). While the generation of influenza-specific T_RMs_ has recently been shown to be vital for robust protection, unlike T_RMs_ generated within the skin or gut (185–187), lung-resident T_RMs_ do not offer long-term protection, rather they require a constant supply of circulating T_EMs_ cells to replenish the niche over time (188) (summarized in Figure 2). In humans, influenza-specific lung-resident T_RM_ cells show a high degree of TCR-sharing with other influenza-specific lung T_EM_ cells, suggesting that both memory cell subsets originate from the same precursors (160). Our understanding of the protective role of memory CD8^+^ T cells in influenza virus infection also comes from experiments with a C57BL/6 mouse model lacking antibodies, where increased numbers of influenza-specific memory CD8^+^ T cells and T_RM_ cells led to markedly reduced influenza-induced morbidity (189). Similarly, primary vaccination with a single-cycle, non-replicative H3N2 IAV induced CD8^+^ T cells capable of protecting against a heterologous (H1N1) lethal challenge (190), an effect that was diminished for mice that had been depleted of CD8^+^ T cells after vaccination. These studies highlight the potential of long-term memory CD8^+^ T cells protecting against severe influenza virus infections. A potential that is not harnessed in the current vaccine strategy.

#### CD8^+^ T Cells Recognize Highly Conserved Influenza Epitopes

CD8^+^ T cells can confer broad cross-protection across different seasonal, pandemic and avian influenza IAV strains due to their ability to recognize relatively conserved viral peptides derived from internal influenza components (NP, M1 and PB1, PB2). The best defined human CD8^+^ T cell influenza epitope is the immunodominant M1_58−66_ peptide bound to the HLA-A^*^02:01 molecule (191–193). This peptide is highly conserved within different influenza A subtypes spanning 100+ years (136), including the 1918 and 2009 pandemic H1N1 strains as well as highly pathogenic H5N1 avian viruses (194). Analysis of immunogenic peptide profiles for the avian H7N9 influenza virus established that it shared six universal CD8^+^ T cell epitopes conserved at ~100% prevalence in human influenza A viruses circulating since the catastrophic Spanish 1918 influenza. These universal human influenza-specific CD8^+^ T cells epitopes include HLA-A^*^02:01/M1_58−66_, HLA-A^*^03:01/NP_265−273_, HLA-B^*^08:01/NP_225−233_, HLA-B^*^18:01/NP_219−226_, HLA-B^*^27:05/NP_383−391_ (although mutants were found in some H3N2 strains) and HLA-B^*^57:01/NP_199−207_ (136). The population coverage by the universal HLAs varies greatly across ethnicities. Fifty-six percent of Caucasians displaying at least one universal HLA, while such coverage reached only 16% in the Alaskan and Australian Indigenous populations (136), highlighting the vulnerability of Indigenous populations toward newly-emerged influenza viruses. Additionally, our recent studies found broadly cross-reactive CD8^+^ T cell responses directed toward the HLA-B37-restricted NP_338_ epitope across IAVs (195), and excitingly, for the HLA-A^*^02:01-restricted PB1-derived epitope across influenza A, B and C viruses (196). The latter introduces a new paradigm whereby CD8^+^ T cells can potentially confer a measure of previously unrecognized cross-reactivity across all human influenza A, B and C viruses, a key finding for the design of universal vaccines.

Influenza-induced morbidity and mortality can correlate with the expression of certain HLAs, including HLA-A^*^24:02, A^*^68:01 or B^*^39:01 alleles, as shown during the 2009 H1N1 pandemic (197). Analysis of peptide scores demonstrated that HLA-A^*^24:02 is more likely to bind variable (rather than conserved) viral regions (197). Similarly, we have previously shown that some HLA alleles, including HLA-A^*^24:02 and A^*^68:01, are less able to elicit robust immune responses toward the highly conserved NP and M1 peptides (136). Both HLA-A^*^24:02 and A^*^68:01, in particular, are found at higher frequencies for Indigenous populations world-wide (136, 197), which may explain the disproportionate impact of pandemic influenza viruses on Indigenous peoples during both (otherwise mild) 2009 pH1N1 pandemic and 1918–1919 (H1N1) Spanish “flu catastrophe” (198–202).

Thus, given the broad potential for cross-protective capacity mediated by CD8^+^ T cells, along with more recent evidence that this effect may indeed be operating in nature to protect people, this aspect of immunity is of considerable interest in terms of developing improved influenza vaccines. However, it is important to note that designing peptide-based T cell vaccines that only cover the major HLA types would clearly be disadvantageous for Indigenous populations globally (203). Further research on CD8^+^ T cell epitopes found in high risk populations is therefore of highest importance to protect people of highest vulnerability.

#### CD8^+^ T Cells Can Confer Broad Cross-Protection for Heterologous IAV Strains

In the context of newly emerging influenza virus infections in people, correlative studies suggest that established CD8^+^ T cell memory confers cross-reactive immunity against severe influenza disease, as observed during the 2009 pandemic H1N1 (pH1N1) outbreak (139, 204). The high (~70%) conservation of CD8^+^ and CD4^+^ T cell epitopes contributing to pre-existing memory may have been a significant factor in the generally mild outcomes of the 2009 H1N1 pandemic (138). Sridhar et al. showed that individuals with higher numbers of CD8^+^ T cells recognizing conserved influenza epitopes fared better following natural infection with the 2009 H1N1 virus (139). The importance of CD8^+^ T cell-mediated immunity was further highlighted in 2013 following the emergence of the novel avian H7N9 strain (205, 206), which killed ~40% of the infected patients. In H7N9-infected individuals, rapid recovery from hospitalization was associated with the presence of significantly more IFN-γ-secreting CD8^+^ T cells when compared to the situation for those who died (207) and recovered (206).

### Development of CTL-Based Vaccines

#### Lessons Learned From the Yellow Fever Vaccine

While the initial experience of IAV infection generally occurs in the first 6 years of life (208), our understanding of both the primary IAV-specific CD8^+^ CTL response and the transition to influenza-specific T cell memory is very limited for humans. Though one paper by Mbawuike et al. reported on primary infection in infants as early as 6 to 13 months of age (209), studies of such influenza exposures in infants are rare, and have not been performed using contemporary approaches for the analysis of T cell-mediated immunity. The closest we have for humans of any age when it comes to the formation of memory CD8^+^ T cells following first virus encounter is for the live-attenuated 17D yellow fever (YF) vaccine. As might be expected from a plethora of mouse experiments, recent YF vaccination studies showed that deuterium-labeled, epitope-specific CD8^+^ CTLs expanded initially following vaccination, before undergoing a contraction phase characteristic of CD8^+^ T cell memory. These vaccine-induced YF-specific memory CD8^+^ T cells persisted in the blood for at least 2 years after YF vaccination, with an average deuterium half-life decay rate of 493 days (210). A similar YF vaccination study in mice demonstrated that, after initial contraction, the long-lived CD8^+^ T cell memory pool remained consistent in size (211), indicating a potential advantage of a CD8^+^ T cell that would need fewer revaccinations compared to the annual recommendation necessary for the seasonal influenza vaccine. Unfortunately for influenza vaccination, the current IIV used in humans does not induce any CD8^+^ T cell responses that can be targeted for such a longevity analysis (115).

#### Vaccination Approaches to Induce Memory CD8^+^ T Cells

Different influenza vaccination approaches are currently being investigated in order to induce long-lasting cross-protective immunity. The only licensed vaccines capable of inducing CD8^+^ T cell immunity, such as the YF vaccine, use live-attenuated pathogens. These are not recommended for influenza “high-risk” groups such as pregnant women, immunosuppressed individuals and the elderly. Therefore, new vaccination strategies need to be developed if we are to protect such vulnerable people. Vogt et al. showed that changing the route of vaccine administration of a quadrivalent inactivated influenza vaccine from intramuscular (i.m.) to transcutaneous induces the expansion of vaccine component-reactive CD8^+^ T cells. Interestingly, the vaccine was also able to induce M1_58−66_-specific responses in a HLA-A^*^02:01-positive donor, although this was only observed in one individual (212). Another approach currently in development is the *Flu-v* CD8^+^ T cell-activating vaccine (213) containing four 21–35 amino acid-long peptides from internal influenza proteins, which can potentially bind to multiple HLA allelic forms, including the highly prominent HLA-A^*^02:01. This approach was protective for HLA-A2 transgenic mice and was also capable of inducing IFN-γ-expressing CD8^+^ T cells across all the participants (*n* = 15) in a phase 1b vaccine trial (213, 214). The *Flu-v* product showed that the vaccine reduces both the viral titer and the symptom score after H3N2 virus challenge in humans (215). However, due to the unknown HLA-restriction of the immunogenic epitopes, the HLA coverage of this vaccine is still to be determined. To circumvent the need for prior knowledge of HLA-restricted epitopes to be included in a universal T cell-based vaccine, particularly for less common HLA allelic variants, full-length influenza proteins have been expressed in Vaccinia virus Ankara vaccine vectors. Berthoud et al. showed that a viral vector encoding for the two internal proteins NP and M1 could induce some CD8^+^ T cell responses (216).

Overall, development of an effective, long-lasting, cross-reactive influenza vaccine relies on an individuals' capacity to generate polyfunctional lung-resident CD8^+^ T cells. However, difficulties in identifying cross-reactive epitopes caused a bottleneck in the development of a universal influenza vaccine. Due to the propensity of IAV to trigger severe outbreaks with pandemic potential, murine models have thus far been developed to test the effectiveness of IAV vaccines based on conserved internal proteins (217–219). While mice immunized with these vaccines can elicit protective CD8^+^ T cell responses, the molecular mechanisms which govern formation of protective memory responses still require further validation in mice, and ultimately in humans.

### Innate and Bystander T Cell Activation During Influenza Virus Infection

In addition to the activation and proliferation of CD8^+^ T cells in a peptide-MHC dependent manner, T cells can also become activated via antigen-independent mechanisms, resulting in proliferation of polyclonal T cells (220). In an influenza mouse model, adoptive transfer of TCR-transgenic OT-I CD8^+^ T cells, which recognizes the ovalbumin peptide, into influenza-infected mice, showed that these OT-I cells can non-specifically expand in the lungs of influenza-infected mice. This suggests that CD8^+^ T cells can become activated independently of their TCRs during primary influenza virus infection (221). Similarly, highly activated CD38^+^HLA-DR^+^ CD8^+^ T cells, numerically greatly exceeding influenza-specific CD8^+^ T cell pools, were found in patients hospitalized with severe H7N9 disease (137), suggesting bystander activation of at least some CD38^+^HLA-DR^+^ CD8^+^ T cells. Despite the evidence that bystander CD8^+^ T cell activation occurs during influenza virus infection, the importance of these cells in terms of viral clearance and the induction of long-term memory is poorly understood. To date, the most solid evidence for the role of bystander activation has been observed in innate-like T cells. These cells, unlike conventional CD8^+^ T cells, recognize non-peptide antigens presented by orthologous MHC I-like molecules. They rapidly secrete cytokines following activation and can mediate some level of protection before adaptive immunity is sufficiently advanced (222). Recently, we demonstrated that mucosal-associated invariant T (MAIT) cells become activated during IAV infections in humans and mice (223, 224). These MAIT cells recognize riboflavin-derivative antigens produced by microbial pathogens (225), but can be variously activated by IL-12/IL-18 (224), IL-15, or type I interferons (226). Using a murine model, we showed that MAIT cells rapidly accumulate and become activated in the infected lung and contribute to protection against IAV infection (223). Similarly, invariant Natural Killer T (iNKT) cells, which recognize lipid antigens presented by CD1d, can protect against murine IAV (227–229). In addition, iNKT cells induced by inactivated influenza A virus vaccination in conjunction with alpha-galactosylceramide, an iNKT cell antigen, can boost influenza-specific memory CD8^+^ T cells and protective immunity in mice (230). The exact contribution of innate T cells vs. conventional CD8^+^ T cell-mediated immunity against influenza viruses is a subject of further investigation. These new insights help to understand the wider range of vaccine responses thus offering us opportunities to generate better strategies to fight against influenza.

## Concluding Remarks

Although current seasonal influenza vaccines can promote the induction of highly specific, long-term memory B cells that produce antibodies against the viral HA1 domain, these antibodies are generally unable to combat newly emerging influenza viruses, including novel pandemic stains and antibody-selected “seasonal” variants that have accumulated mutations in those epitopes surrounding the receptor binding pocket. Generation of high-affinity neutralizing Abs against conserved surface epitopes remains a constant challenge to provide long-lasting and cross-protective B cell memory, and as such, more work is needed to better understand B cell responses against natural infection vs. vaccination, in order to design better B cell- or antibody-based universal vaccines. On the other hand, an influenza vaccine capable of stimulating CD8^+^ T cell responses would generate long-term T cell memory against conserved epitopes without the need for annual vaccination. In addition, a role for innate-like T cells in influenza protection is increasingly emerging, which could potentially be important both for the development of novel therapeutics and for boosting (or maintaining) long-term memory. As a consequence, substantial efforts are being made globally to exploit both innate and adaptive immune components for the development of novel influenza vaccines that induce long-lasting B cell/antibody and/or cross-reactive T cell immune memory populations.

## Author Contributions

MA and AF wrote the sections on influenza and humoral immunity. LH and XJ wrote the sections on cellular immunity. BC wrote the section on vaccine adjuvants. TN, KK, and PD wrote and modified the original manuscript, and the revised versions.

### Conflict of Interest Statement

The authors declare that the research was conducted in the absence of any commercial or financial relationships that could be construed as a potential conflict of interest.
